# Time perception and patience: individual differences in interval timing precision predict choice impulsivity in European starlings, *Sturnus vulgaris*

**DOI:** 10.1007/s10071-020-01456-2

**Published:** 2021-01-12

**Authors:** Clare Andrews, Jonathon Dunn, Daniel Nettle, Melissa Bateson

**Affiliations:** 1 Department of Psychology, University of Stirling, Stirling, UK; 2 Centre for Behaviour and Evolution, Newcastle University, Newcastle upon Tyne, UK; 3 Centre for Behaviour and Evolution and Newcastle University Biosciences Institute, Newcastle University, Newcastle upon Tyne, UK; 4 Centre for Behaviour and Evolution and Population Health Sciences Institute, Newcastle University, Newcastle upon Tyne, UK

**Keywords:** Interval timing, Scalar expectancy theory, Impulsivity, Delay discounting, Starlings, *Sturnus vulgaris*

## Abstract

Impulsivity, in the sense of the extent rewards are devalued as the time until their realization increases, is linked to various negative outcomes in humans, yet understanding of the cognitive mechanisms underlying it is limited. Variation in the imprecision of interval timing is a possible contributor to variation in impulsivity. We use a numerical model to generate predictions concerning the effect of timing imprecision on impulsivity. We distinguish between fixed imprecision (the imprecision that applies even when timing the very shortest time intervals) and proportional imprecision (the rate at which imprecision increases as the interval becomes longer). The model predicts that impulsivity should increase with increasing fixed imprecision, but decrease with increasing proportional imprecision. We present data from a cohort of European starlings (*Sturnus vulgaris*, *n* = 28) in which impulsivity had previously been measured through an intertemporal choice paradigm. We tested interval timing imprecision in the same individuals using a tri-peak temporal reproduction procedure. We found repeatable individual differences in both fixed and proportional imprecision. As predicted, birds with greater proportional imprecision in interval timing made fewer impulsive choices, whilst those with greater fixed imprecision tended to make more. Contradictory observations in the literature regarding the direction of association between timing imprecision and impulsivity might be clarified by distinguishing between fixed and proportional components of imprecision.

**Supplementary Information:**

The online version contains supplementary material available at 10.1007/s10071-020-01456-2.

## Introduction

The term impulsivity is used in a variety of senses (Evenden 1999). Here, we use it to mean the extent by which a reward is devalued as the time to its realization increases, also known as delay discounting. Impulsivity of this kind varies between individuals. In humans, greater impulsivity has been linked to psychological conditions ranging from behavioural disorders to addiction (Moeller et al. 2001; Verdejo-García et al. 2008; Patros et al. 2016). In non-human animals, impulsivity is influenced both by current energetic state, and long-term developmental history (Bateson et al. 2015; Dunn et al. 2019). However, in neither humans nor non-human animals do we fully understand the cognitive mechanisms that underlie individual differences in impulsivity. These mechanisms are likely to involve the processing of reward magnitude, or the processing of time intervals, or both.

The idea that core cognitive processes relating to time might be different in more impulsive individuals has generated a substantial literature in humans (see Wittmann and Paulus 2008), though there is at present no consensus on exactly what feature of time processing drives variation in impulsivity. Several recent rat studies have found correlations between impulsivity and the imprecision of timing in particular. Imprecision is the amount of variation (noise) when an individual repeatedly estimates the same time interval; it is thus synonymous with the ‘endogenous variability’ discussed by Balci et al. (2009). Two correlational studies found that those individuals whose timing was more imprecise were also more impulsive (Marshall et al. 2014; McClure et al. 2014). In these studies, impulsivity was measured by repeated choices between smaller sooner and larger later rewards, and timing imprecision was measured by either repeated reproduction of a fixed interval (McClure et al. 2014), or a temporal discrimination task (Marshall et al. 2014). In the former case, animals are trained that a response is available after a fixed interval (FI). The imprecision of their timing is estimated by the temporal spread of their responses about the trained interval. In the latter case, animals are trained to discriminate between a short and a long duration stimulus, then tested on a series of stimuli of intermediate durations. The shape of the logistic function mapping their probability of responding ‘long’ onto the duration of the stimulus provides an estimate of timing precision.

A third rat study (Smith et al. 2015) presents three separate experiments where impulsivity and timing imprecision were measured simultaneously, and then both measured again in the same individuals after an intervention phase. The intervention phase, which varied in detail across the three experiments, consisted of prolonged working on a task requiring the animal to make responses at exact points in time, such as a differential reinforcement of low rates of responding schedule. In all cases, not only had timing imprecision decreased relative to baseline when it was measured again after the intervention phase; the rats had become less impulsive too. Thus, these results suggest that timing imprecision and impulsivity are sufficiently closely linked that the same interventions that affect one also affect the other.

Whilst these three rat studies suggest empirically that timing imprecision might be related to impulsivity, they have not formally theorized why this relationship should hold. Intuitively, timing imprecision and impulsivity seem like they should be related. There is a non-linear, convex downward function linking the perceived value of a reward to the delay until its realization. An individual with an imprecise perception of, say, an 8 s delay will sometimes perceive that delay as 7 s, and sometimes as 9 s. Because of the convexity of the discount function, the individual will overestimate the perceived value of the reward when it perceives the delay to be 7 s by a greater amount than it will underestimate the perceived value when it perceives the delay to be 9 s. Thus, its average valuation of the reward will be greater than if its timing were completely precise, even though its average estimate of the delay is 8 s. This is an example of Jensen’s inequality (Jensen 1906; Denny 2017). The magnitude of the over-valuing effect, for any single option, can be predicted provided one knows the shape of the discount function and the discount rate *k* (Mazur 2004).

This principle has been used to make the prediction that greater timing imprecision should produce less impulsivity (McClure et al. 2014). The study in fact found the opposite pattern. However, we argue that the prediction is not so straightforward. Impulsivity is typically measured using choices between smaller sooner and larger later rewards. Imprecision of interval estimation will apply both to the estimation of the short delay to the smaller sooner reward, and the long delay to the larger later reward. It is not intuitively obvious which of the two options will be more strongly overvalued because of the imprecision (or indeed, whether the overvaluations of the two options will exactly cancel one another out).

**Fig. 1:**
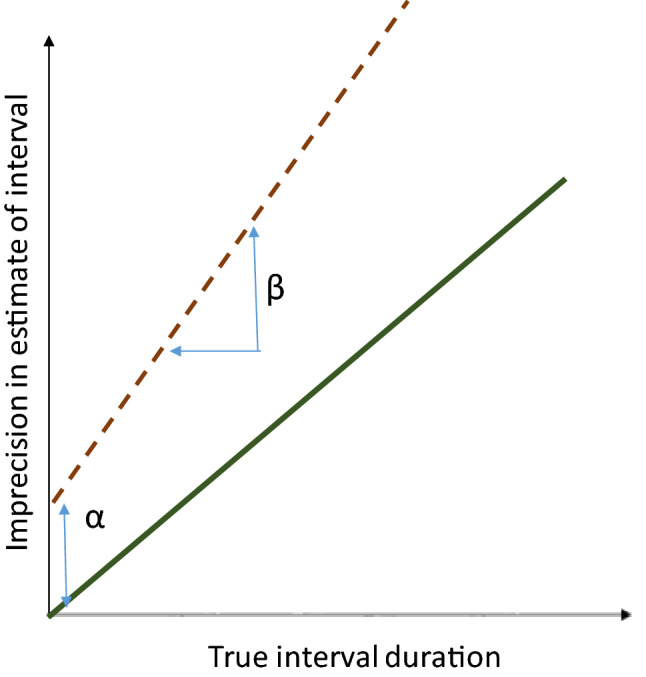
Two components of individual differences in timing imprecision. The imprecision in interval timing increases with the interval to be timed. The solid and dashed lines show this relationship for two hypothetical individuals. Here, one individual (dashed line) is more imprecise than another (solid line), in terms of a larger intercept of the line describing the magnitude of imprecision for a given interval duration (*α*, fixed imprecision), and also in terms of this line having a steeper slope (*β*, proportional imprecision)

Saying that one individual’s interval timing is less precise than that of another individual could mean two different things (Fig. 1): (a) the imprecise individual’s variance in perceived delay exceeds that of the precise individual by some constant that is independent of the delay duration; or (b) the imprecise individual’s variance in perceived delay increases more steeply with increasing delay than is true for the precise individual. If we model timing imprecision as a linear function of the delay to be estimated, then these two forms of imprecision amount to differences in the intercept (α on Fig. 1) of the imprecision function (i.e. the variance in estimation of even a very short delay), and the slope (β on Fig. 1) of the imprecision function. We henceforth refer to the intercept of the imprecision function as the fixed imprecision, and the slope of the imprecision function as the proportional imprecision. It remains to be determined which of the fixed and proportional imprecision ought to affect impulsivity, or whether their effects are even in the same direction. We could find no analytical framework in which to make predictions on this question. We, therefore, developed a numerical model. We then tested the model’s predictions using data from a cohort of European starlings.

## Numerical model: how should timing imprecision affect impulsivity?

Our numerical model was written in R (R Core Development Team 2018). The code implementing it is available in the Zenodo data repository (https://zenodo.org/record/3971670). In the model, individuals discount rewards that arrive after a delay *t* according to the hyperbolic discounting function (Mazur and Biondi 2009):

$$v\left( t \right) = \frac{1}{{\left( {1 + kt} \right)}}$$

Here, *k* is a parameter setting the discount rate. We assume that when an individual experiences a delay, their timing of it will on average be accurate, but with some imprecision. Specifically, for individual *i*, the perceived duration of an interval of true duration *t* is normally distributed as:

$$p_{i,t} \sim N\left( {t, \alpha_{i} + \beta_{i} t} \right)$$

Here, ${\alpha }_{i}$ is individual *i*’s fixed imprecision, and ${\beta }_{i}$ is their proportional imprecision. Scalar expectancy theory states that timing imprecision increases with increasing interval duration, with constant ratio between the mean estimate of the duration and its variance (the scalar variance property; Gibbon 1977). Strictly, under scalar variance, fixed imprecision should be zero, whilst proportional imprecision should be a positive constant. However, scalar variance does not hold exactly: even in the original studies, there was a small but positive intercept of the regression line of imprecision on FI (Gibbon 1991). This has typically been treated as unimportant noise, and individual differences in this fixed component of imprecision have not been separately characterised. However, it is reasonable to assume that both *α* and *β* typically have positive values and can vary between individuals.

We simulated the scenario where there are two rewards, one that arrives after 3 s and one that arrives after 8 s. Individuals assign a value to each outcome based on the perceived rather than the true delay to reward. Their value for the option after experience is the mean of the values of the individual instances of it they have experienced. Thus, for each option, our model implements Mazur’s (2004) equation for predicting the value of a reward where there is variability in delay. The parameter of interest is the relative value assigned to a smaller sooner reward compared to a larger later reward (averaged over 100,000 experiences of each delay, and normalized relative to the values assigned by an individual with perfectly precise timing, i.e. $\alpha =0$ and $\beta =1$). Hence, a higher relative value equates to greater impulsivity. We explore the effects of varying the individual’s values of *α* and$\beta$.

**Fig. 2:**
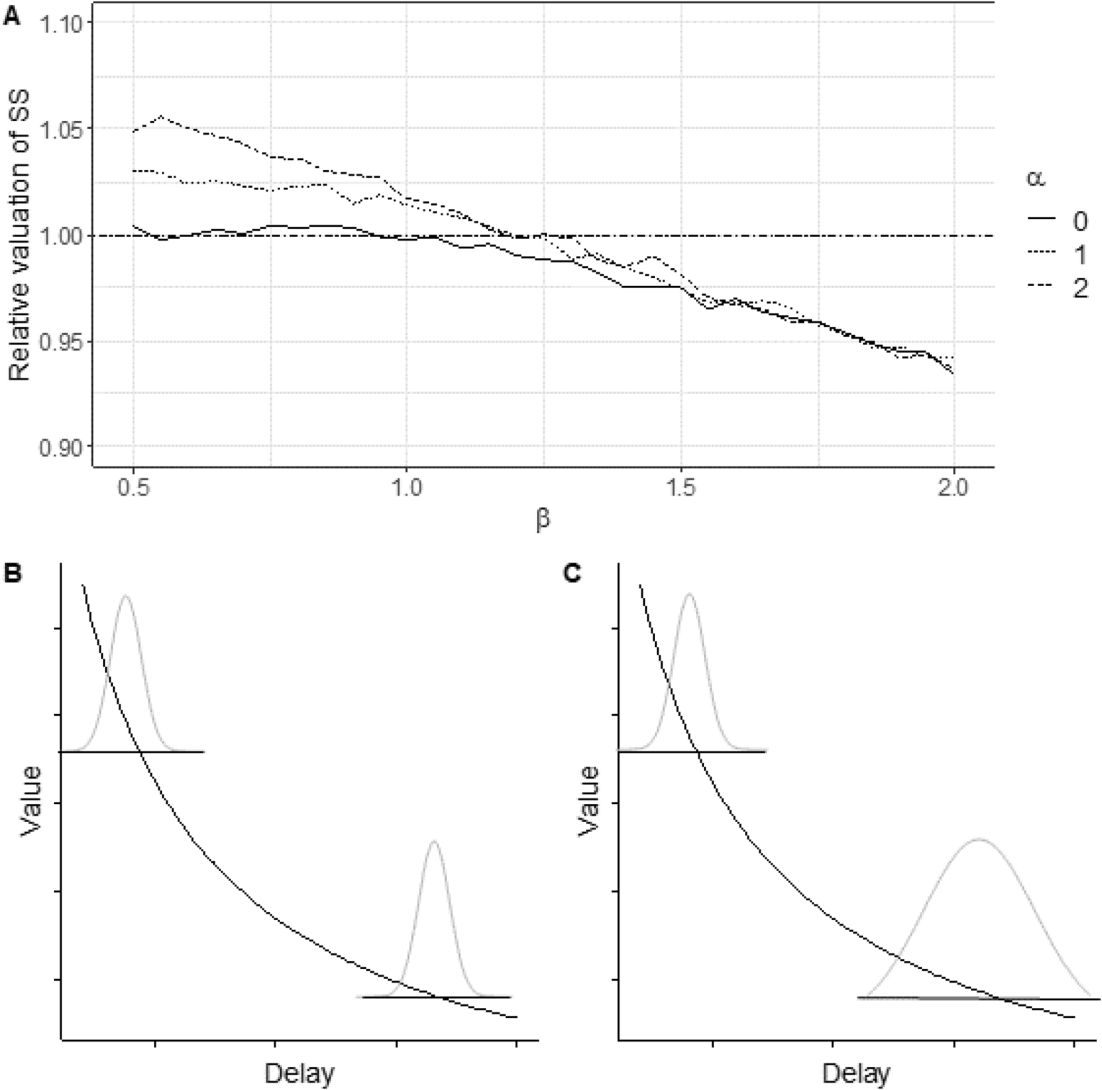
**a** Predicted effect from the numerical model of changes in the fixed component of timing imprecision (*α*) and the proportional component of timing imprecision (*β*) on the relative valuation of a smaller sooner reward (SS) after 3 s and a larger later reward after 8 s. Valuations are relative to those of an individual with perfectly precise timing. A higher relative valuation would lead to greater impulsivity. The discount rate is set at *k* = 0.54, an empirically derived estimate for starlings. **b**, **c** Illustrative explanations of the effects predicted by the model. **b** Additional imprecision that has the same magnitude at all delays (i.e. in *α*) has a stronger over-valuing effect on the smaller sooner reward than the larger later reward, because the discount function is steeper at this point. **c** Additional imprecision whose magnitude is proportional to the delay (i.e. in *β*) has a stronger over-valuing effect on the larger later reward than the smaller sooner reward, because the additional imprecision is greater at the longer delay

Results are shown in Fig. 2. We use *k* = 0.54, the average discount rate estimated from a previous study of European starlings (Bateson et al. 2015). Where the proportional imprecision $\beta$ is small, increasing the fixed imprecision *α* produces greater impulsivity. This is intuitive: if there is a constant increment in imprecision that applies to the estimation of both rewards, then its over-valuation impact will be greater on the smaller sooner reward, because the discount function is steepest at this point (for a graphical illustration, see Fig. 2b). Thus, it will produce greater over-valuation of that reward and greater impulsivity. However, for any given *α*, increasing the proportional imprecision $\beta$ *reduces* impulsivity. This is because an excess of proportional imprecision will lead to larger error in the estimation of the delay to the larger later reward in particular, producing a greater over-valuation of that reward, and hence less impulsivity (Fig. 2c). As $\beta$ becomes larger, the differences in impulsivity with increasing *α* disappear. With 100,000 samples at each delay, these results show very little variation from run to run of the simulations. We also repeated the simulations with both higher and lower discount rates. Results were qualitatively similar, though with very low values of *k*, sensitivity to variation in first *α* then $\beta$ is abolished. This is because, in the limit as $k\to 0$, the hyperbolic discount function becomes a horizontal straight line, and Jensen’s inequality does not apply.

The model shows that we cannot make a global prediction concerning the association between timing imprecision and impulsivity. Instead, timing imprecision must be decomposed into the fixed component (the component of imprecision that is independent of the delay), and the proportional component (the rate by which imprecision increases as the delay increases). The fixed component of imprecision should either be positively associated with impulsivity (greater fixed imprecision, more impulsive; as long as the proportional component is relatively small), or that the association should be null (if the proportional component is large). The proportional component of imprecision should be negatively associated with impulsivity (greater proportional imprecision, less impulsive).

In the remainder of this paper, we test these predictions using data from a cohort of European starlings. We measured interval timing using a tri-peak procedure (Hinton and Meck 1997; Paule et al. 1999). Because this involves reproduction of three different interval durations, the tri-peak procedure allows us to separately estimate the fixed and proportional imprecision for each individual. We had a prior measurement of impulsivity from the same birds, based on an intertemporal choice task (Dunn et al. 2019). The birds, which were hand reared, had undergone a manipulation of developmental conditions that affected their impulsivity (see Dunn et al. 2019 for details). Specifically, individuals raised under the combination of limited food supply and high begging effort were significantly less impulsive than other experimental groups. We thus predicted: (1) that birds with greater fixed imprecision in the timing task would if anything make more impulsive choices in the impulsivity task; (2) that birds with greater proportional imprecision in the timing task would make fewer impulsive choices in the impulsivity task; and (3) that the developmental factors that affected impulsivity would also affect timing imprecision.

## Methods

### Ethics

Our study adhered to the ASAB/ABS Guidelines for the ethical treatment of animals, was approved by Newcastle University Animal Welfare Ethical Review Board committee and was conducted under U.K. Home Office project licence (number PPL70/8089).

### Study animals and developmental manipulation

Subjects were 28 European starlings (15 female, 13 male; sexed molecularly; see Nettle et al. 2017) from 8 natal families belonging to a cohort of 32 chicks hatched in the wild in May 2014 in a nest box population on farms in Northumberland, UK. Three of the cohort had died prior to the present experiment and a further bird failed to complete the tri-peak procedure task; impulsivity data had been successfully obtained for 27 of the 28 birds.

As nestlings, the birds were subject to a manipulation of developmental adversity described in full elsewhere (Nettle et al. 2017). Briefly, on post-hatching day 5, quartets of siblings were brought to the lab where they were hand reared. On day 6 and continuing until day 15, we simultaneously manipulated amount of food (hereafter Amount: Plenty or Lean) and the begging effort (Effort: Easy or Hard) experienced by the nestlings, in a 2 × 2 factorial design. Nestlings allocated to the Plenty groups were fed ad libitum to satiation at each of nine feeding visits per day, while the Lean groups received a proportion of the amount consumed by the corresponding Plenty group. Initially, this proportion was set to 70%, but it was dynamically adjusted each day so that the weight gain of the Lean birds tracked that of the lightest nestlings in a previous study of wild-reared nestlings. The total amount fed to the Lean groups over the whole manipulation was 72.75% that of the Plenty groups. Thus, each Lean group received a smaller amount of food over the developmental period than the corresponding Plenty group, whilst begging for a similar amount of time each day Lean birds were lighter at fledging and later to fledge than Plenty birds, and were permanently skeletally smaller than the Plenty birds (Nettle et al. 2017). To manipulate begging Effort, nestlings in the Hard groups received, in addition to the nine feeding visits per day, nine sham feeding visits. During the sham visits, nestlings were stimulated to beg for 2 min (the approximate duration of a feed) without receiving food. Thus, each Hard group received a similar amount of food as the corresponding Lean group, but had to beg around twice as much to receive it. Hard birds maintained lower body weights than Easy Birds through adulthood, and showed evidence of different foraging strategies (Dunn et al. 2018).

From day 16 onwards, all birds received ad libitum food. Once the fledglings became independent (approx. 4 weeks post-hatch), they were transferred into mixed-sex, mixed-treatment groups housed in two indoor aviaries (215 × 340 cm and 220 cm high; ca. 18 °C; 40% humidity; 15:9 h light:dark cycle) and were fed ad libitum on domestic chick crumb (Special Diets Services ‘Poultry Starter (HPS)’), supplemented with cat biscuits (Royal Canin Ltd.), dried insect food (Orlux insect pâté), live mealworms and fruit.

### Experimental cages

For both the timing and impulsivity experiments, birds were transferred to individual cages that served both as home cages and operant testing chambers. Cages measured 100 × 45 cm and 45 cm high and were fitted with two wooden perches and two water bottles. Each cage contained an operant panel of three illuminable pecking keys and a feeder trough connected to a pellet dispenser delivering 45-mg grain-based rodent pellets (TestDiet, Richmond, IN, USA), as described in Feenders and Bateson (2013). The panels were controlled remotely using the Whisker Experimental Control system (Cardinal and Aitken 2010), and cognitive tasks were programmed in Microsoft Visual Basic 5.0 (Microsoft Corporation, Redmond, WA, U.S.A). Temperature and lighting conditions were the same in the experimental room as in the aviary. There were eight cages in the experimental room, with acoustic and visual contact between them. Due to the number of cages available, the birds were run in sequential groups of 6–8 birds for both the impulsivity and timing tasks.

### Impulsivity task

Data on impulsive choice have been reported fully elsewhere (Dunn et al. 2019). Impulsive choice testing began when birds were aged 978–1044 days. Briefly, birds made repeated simultaneous choices between a smaller sooner and a larger later food reward, using the same operant apparatus as above. At the start of each trial, the central pecking key was illuminated with amber light, and a single peck to this key was required to initiate the trial, whereby the amber light extinguished and either a forced or choice trial began. In a forced short-delay trial, pecking a side key illuminated in red produced one 45 mg pellet after a 3 s delay. In a forced long-delay trial, pecking a side key illuminated in green produced two pellets after an 8 s delay. Choice trials were identical to forced trials with the exception that following the initiation peck, both side keys were illuminated (one in red and one in green). A single peck indicated the bird’s choice. Birds completed 120 trials (or a maximum of 4 h) per day, 7 days a week and all completed 10 days of the impulsivity task. We used the proportion of completed choice trials on which the bird chose the smaller sooner key as the measure of impulsivity using data for each bird from day 5 to day 10 of the task inclusive.

### Timing imprecision: tri-peak procedure temporal reproduction task

The tri-peak procedure task began when birds were 1308–1658 days old. Habituation and operant training procedures followed those described by Dunn et al. (2019). To progress on to the tri-peak procedure task, birds had to peck on at least 80% of initial training trials involving one lit key for three consecutive sessions, or at least 50% of trials for five consecutive sessions. Trials took place between 0730 and 1230, with ad libitum food (10 g dry cat biscuits, 5 g chick crumb, 5 g dried insect food, a slice of fruit and 4 live mealworms) and water baths provided from 1230 until 1630.

Birds were trained on three fixed-interval (FI) reinforcement schedule durations (5 s, 15 s and 45 s) associated with three separate pecking keys. Since we were primarily interested in individual differences and hence required all birds to have the same experience, all birds received the same assignment of key positions to intervals, namely: left key to 5 s, central key to 15 s and right key to 45 s. At the start of each trial, the central pecking key was illuminated with amber light. This remained illuminated until the bird initiated a trial by a single peck to this key, whereby the amber light extinguished and all three pecking keys were illuminated in green. On SHORT trials, a single peck to the left key after an interval of 5 s or greater caused all keys to extinguish and initiated the illumination of the hopper light and delivery of one reward pellet. On MEDIUM trials, a single peck to the central key after an interval of 15 s or greater was rewarded with a pellet. On LONG trials, a peck to the right key after an interval of 45 s or greater was rewarded with a pellet. There were no consequences of responses on incorrect keys, or early responses on the correct key. An inter-trial interval (ITI) of 100 s began following pellet delivery. Each daily session comprised 2 blocks (beginning 07:30 and 10:10), each with a maximum of 30 trials and ending after 2.5 h if a bird had not completed. Within each block, an equal number of SHORT, MEDIUM and LONG trials were presented in random order.

Once a bird completed at least 80% of trials each day for 5 consecutive days, the 30 SHORT, MEDIUM and LONG trials of each block were randomly interspersed with 10 PROBE trials. In PROBE trials, all keys remained illuminated for 135 s and then extinguished, with no consequences for pecks on any key, and no reward given. We recorded the number of peck responses on all keys throughout all trials in 0.5 s time bins from the initiation of each trial. Birds were tested 7 days a week and completed between 873 and 2026 trials in total, the procedure ending when all birds in each group had completed at least 9 days with PROBE trials (range 9–22 days). This large number of trials provided birds with opportunity to learn the FI durations and develop stable responses. We analysed only data from PROBE trials on the final 4 days of the task (i.e. a maximum of 60 PROBE trials per bird).

### Statistical analysis

The raw data and R script are archived in the Zenodo repository (https://doi.org/10.5281/zenodo.3971670). Statistical analyses were conducted in R v3.5.1 (“R Development Core Team” 2011) using the base statistical procedures and ‘lme4’ packages (Bates et al. 2015).

Under FI schedules, animals tend to begin responding at a low rate and increase response frequency as the criterion FI approaches. In PROBE trials, where no reward is delivered, response frequencies taper off once the FI has passed. Thus, we described birds’ timing functions for each of the three FIs using three parameters (Hinton and Meck 1997): (1) Peak Rate—the maximal rate of pecking on the target key, a measure of motivation; (2) Peak time (hereafter Peak)—the time of the maximal rate of pecking, a measure of the bird’s central estimate of the FI; and (3) Spread—which we here define as the duration between the lower bound and upper bound time at which the peck rate was half the Peak Rate. This is a measure of timing imprecision, with a larger Spread indicating greater imprecision (Church et al. 1994).

There are two approaches to estimating Peak Rate, Peak and Spread (Church et al. 1994). The mean-response approach first averages responses across trials, and then estimates the target parameters for each animal from those average response patterns. The single-trials approach estimates Peak Rate, Peak and Spread from each trial, and only then averages those estimates to give a value for each animal. Using the data from the first 14 birds to be run, we experimented with the single-trials approach. This assumes that each trial can be characterized by an initial period at one rate of responding, a discrete step up to a higher rate as the FI approaches, and a discrete step down again to a lower rate after the FI has passed. The temporal positions of the steps up and down for each trial are estimated numerically from the pattern of responses. However, if responses occur in bursts, the two-step model fits poorly and/or produces step-points that fail to include most responses within the high-rate period. For this reason, researchers using the single-trials approach exclude some trials, using various fit criteria (Church et al. 1994; Matell et al. 2006). In the present case, responses often occurred in bursts, especially in the longest FI, as birds returned briefly to their perches during the trials. Thus, applying the single-trials approach resulted in the exclusion of a high proportion of trials, whichever fit criteria we applied.

Instead, we used the mean-response approach. For each bird, we calculated the mean number of responses on each key within each 0.5-s time bin across all PROBE trials during the final 4 days of the task. Next, we fitted polynomial functions to these data for each bird. Using data from the first 14 birds, we systematically increased the order of the polynomial functions used, from two upwards. The proximity of the Peak estimated from the polynomial function to the numerical maximum of the data (and to the duration of the trained FI) continued to improve as the order of the polynomial increased, particularly for the shortest FI, but with little further gain above order 10. We thus used 10th-order polynomials for all subsequent estimation. From the fitted polynomials, we defined the Peak as the time of the maximum fitted value. Peak Rate was defined as the rate of pecking corresponding to the Peak. We then defined as the lower and upper bounds the time at which peck rate equaled half the Peak Rate, and calculated the Spread as defined above. Having obtained, for each bird, a Spread value for each of the FIs, we fitted a linear model with the FI duration as the predictor variable and the Spread as the outcome. The intercept from this model was our estimate of that bird’s fixed imprecision, and the slope was our estimate of the bird’s proportional imprecision.

For the main analyses, we used general linear mixed models incorporating random intercepts for natal family, and individual bird where we had repeated measures. The fixed effects included in each model are described in the relevant Results section and in Table 2. We included sex as a control variable in all analyses of timing performance measures to allow for the detection of sex-specific effects, because sex differences in interval timing occur in rats (McClure et al. 2014); results without controlling for sex are extremely similar. For models including the developmental treatments, we initially included interaction terms (e.g. Amount*Effort) and sequentially removed non-significant interactions to produce the final models reported in Table 2. Measures of Peak and Spread were, where appropriate, normalised across FI durations prior to analysis by standardizing them to the mean and standard deviation of that FI. Fixed and proportional imprecision were standardized for analysis but not for plotting. Maximum-likelihood estimation was employed throughout. Significance testing for mixed models used Satterthwaite’s method in R package ‘lmerTest’. We assumed a criterion for significance of *p* < 0.05.

To examine individual consistency in timing performance measures, we separately fitted polynomials on data from PROBE trials on the final two days of the tri-peak procedure and the 2 days prior to this, and conducted a variance components analysis to ascertain the proportion of variance explained by individual identity and natal family in fixed and proportional imprecision.

## Results

### Overall timing performance

**Fig. 3:**
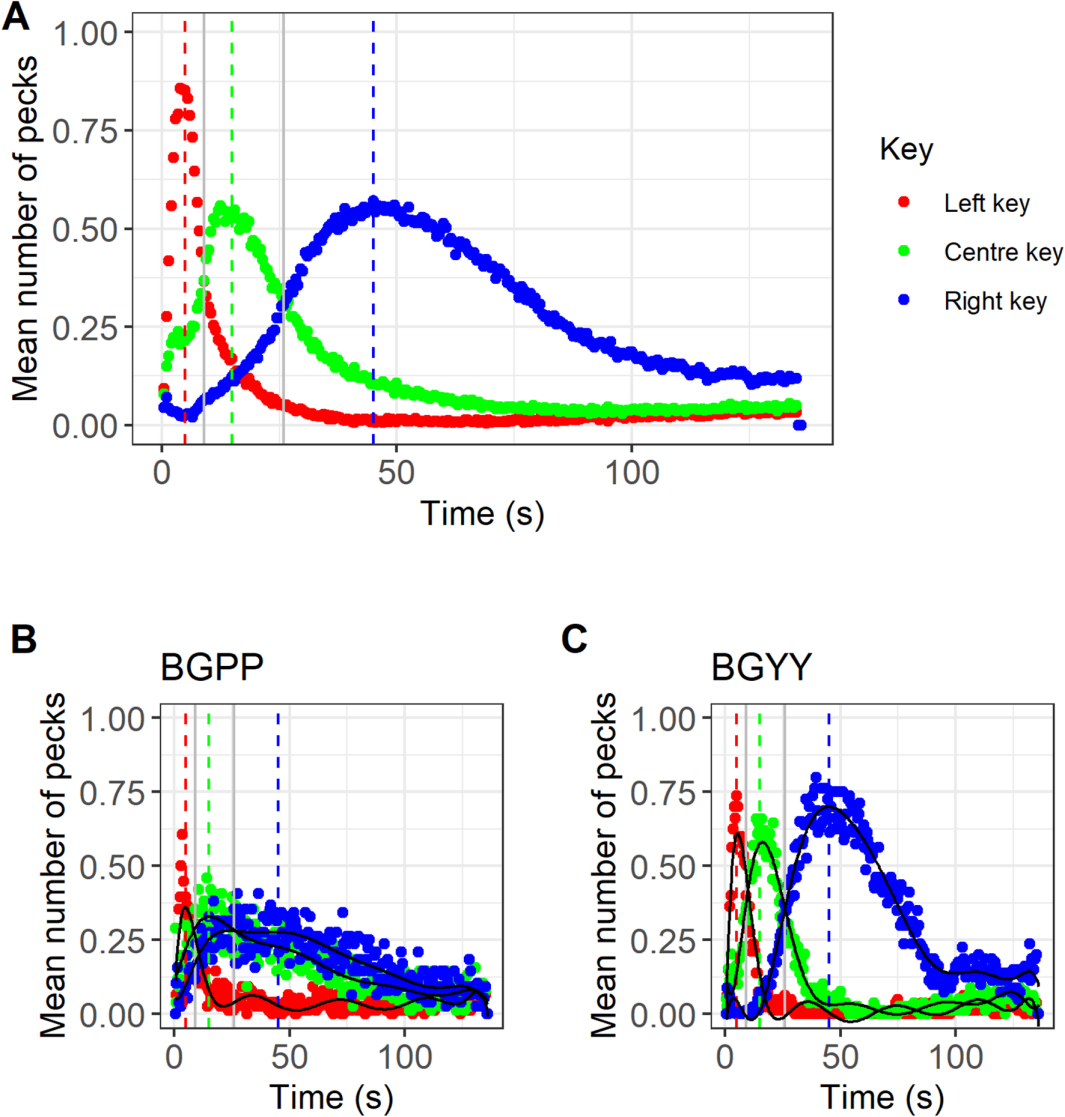
**a** Overall tri-peak temporal reproduction procedure performance averaged across all birds. **b**, **c** Plots from two individual birds with differing interval timing performance. Fitted polynomial shown (solid line). The individual (BGPP) in panel **b** shows greater imprecision (Spread) as compared to the individual (BGYY) in panel **c**. Data show the mean number of responses on each key in 0.5 s time bins during PROBE trials during the final 4 days of the task. Vertical dashed reference lines show the FI durations on which birds were trained. Vertical solid grey lines indicate the geometric means of the first and second FI durations, and the second and third FI durations. Data for the remaining 25 birds are shown in Supporting Information, figure S2

**Fig. 4:**
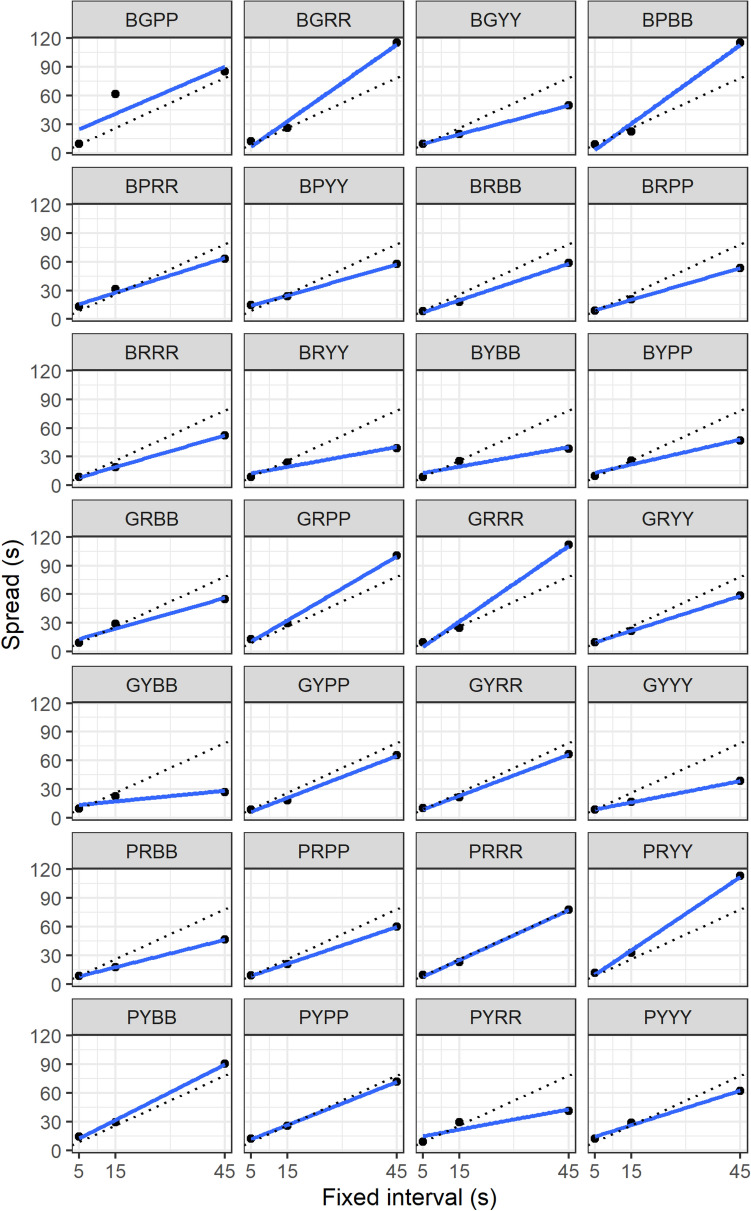
Spread of responses against fixed interval duration, by bird. Data are from the final 4 days of the tri-peak temporal reproduction task. Individual starlings are identified by a 4-letter code. Solid lines represent linear fits for each bird. Dotted lines represent expectation under the assumption of scalar variance, with the ratio of Spread to FI delay given by the grand mean across birds and delays

Key pecking responses on each key were distributed across time with a single peak, as expected (Fig. 3). On average, birds showed accurate timing, with mean Peak values falling close to the trained FIs (mean ± SE, SHORT (5 s FI) 4.77 ± 0.18 s; MEDIUM (15 s FI) 14.21 ± 0.60 s; LONG (45 s FI) 41.38 ± 1.72 s; Fig. 3). Birds switched their highest response rates from key to key at the geometric means of the two trained FIs: left to centre, geometric mean 8.66 s, observed 9 s; centre to right: geometric mean 25.98 s, observed 26.5 s. As expected, average Spread increased with FI for all birds (mean ± SE, SHORT (5 s FI) 10.27 ± 0.36 s; MEDIUM (15 s FI) 25.34 ± 1.58 s; LONG (45 s FI) 66.45 ± 4.82 s; see Fig. 4 for data from each bird). However, the birds’ performance did not show the scalar variance property. The scalar variance property implies a constant coefficient of variation across FIs (i.e. the Spread divided by the FI should be constant across SHORT, MEDIUM and LONG keys). In fact, Spread/FI was significantly higher for SHORT (mean ± SE 2.05 ± 0.07) than MEDIUM (mean ± SE 1.69 ± 0.11) or LONG (mean ± SE 1.48 ± 0.11; linear mixed model: *β*_MEDIUM_ = −0.22, SE 0.06, *t* = − 3.65, *p* < 0.001; *β*_LONG_ = − 0.38, SE 0.06, *t* = − 6.43, *p* < 0.001; figure S1). This implies the existence of fixed as well as proportional imprecision in the birds’ timing performance. Indeed, birds displayed non-zero fixed imprecision overall (mean ± SE: 3.76 ± 1.18 s, *t* test against *μ* = 0, *t* = 3.19, *p* = 0.004) as well as non-zero proportional imprecision overall (mean ± SE: 1.40 ± 0.12 s, *t* test against *μ* = 0, *t* = 11.28, *p* < 0.001).

Seven birds had fixed imprecision values of less than zero. Negative imprecision is impossible, and thus this indicates imperfect estimation of the underlying parameter of interest. On the principle of minimising data manipulation, we not only retained the negative values, but also calculated a truncated version of the variable where negative values were set to zero. We repeated the analyses with the truncated version, and report these briefly below where relevant.

Birds with greater fixed imprecision showed lower proportional imprecision (*r* = − 0.79, *p* < 0.001; this correlation was slightly attenuated using the truncated fixed imprecision variable, *r* = − 0.62, *p* < 0.001). We also calculated the correlations of fixed and proportional imprecision to the Spreads at each of the three FIs (Table 1). Fixed imprecision was unrelated to the Spread at 5 s, significantly positively related to the Spread at 15 s, and significantly negatively related to the Spread at 45 s. Proportional imprecision was almost perfectly correlated with the Spread at 45 s. Thus, the hallmark of a bird with high proportional imprecision is that they are more imprecise at the longest delay.

**Table 1:** Correlations (*r*) between fixed and proportional imprecision values, and the spread of the fitted polynomial at each of the three FI delays

	Spread 5 s	Spread 15 s	Spread 45 s
Fixed imprecision α	− 0.01	0.41*	− 0.71*
Proportional imprecision β	0.35	0.22	0.99*

Results are qualitatively similar using the truncated version of the fixed imprecision variable

*p < 0.05

### Timing imprecision and impulsivity

**Fig. 5:**
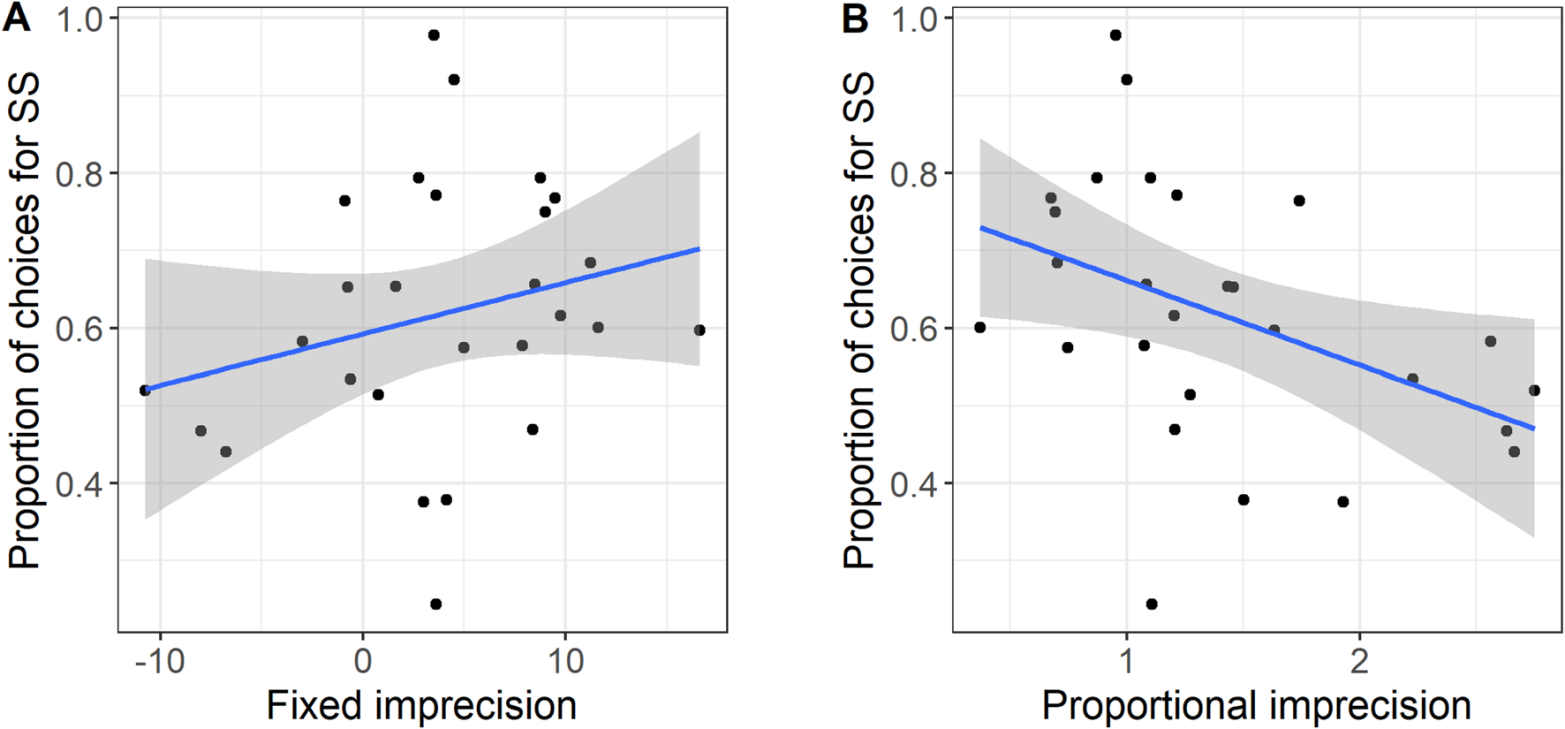
**a** Relationship between fixed imprecision in timing and the proportion of choices for the smaller sooner reward (SS) in the impulsivity task. **b** Relationship between proportional imprecision in timing and the proportion of choices for the smaller sooner reward (SS) in the impulsivity task. Lines represent linear fits, and shaded areas 95% confidence intervals

To examine the relationship between choice impulsivity and interval timing precision, we ran two models with proportion of choices for the smaller sooner reward as the outcome variable, and fixed or proportional imprecision as the predictor variables (Table 2, models 1 and 2). The high degree of collinearity between fixed and proportional imprecision prevented them being entered simultaneously into the same model (see “Discussion”). The association between fixed imprecision and impulsivity was non-significantly positive (Table 2, model 1; Fig. 5a; using the truncated version of this variable instead produced the same conclusion and a slightly smaller but still positive parameter estimate). Proportional imprecision significantly predicted choice impulsivity, negatively, with more imprecise birds making fewer choices for the smaller sooner reward (Table 1, model 2; Fig. 5b).

**Table 2:** Output of mixed models. SS: smaller sooner

Model	Response variable	Fixed predictor variables	Random effects	*B* (SE)	*t*	*p* value	*n*
Choice impulsivity							
1	Proportion SS choices	Fixed imprecision	Natal family	0.06 (0.03)	1.81	0.083	27
		Sex: M		0.05 (0.06)	0.78	0.443	
2	Proportion SS choices	Proportional imprecision	Natal family	− 0.10 (0.03)	− 3.62	0.001	
		Sex: M		0.05 (0.05)	1.03	0.313	
Interval timing precision							
3	Fixed imprecision	Amount: Lean	Natal family	− 0.16 (0.39)	− 0.40	0.692	28
		Effort: Hard		− 0.41 (0.39)	− 1.06	0.300	
		Sex: M		− 0.72 (0.43)	− 1.67	0.106	
4	Proportional imprecision	Amount: Lean	Natal family	0.65 (0.38)	1.72	0.101	28
		Effort: Hard		0.45 (0.38)	1.19	0.247	
		Sex: M		0.06 (0.42)	0.14	0.887	
Exploratory models							
5	Spread (standardised by key)	Amount: Lean	Natal family/Bird	0.55 (0.23)	2.33	0.030	84
		Effort: Hard		0.18 (0.24)	0.76	0.457	
		Sex: M		− 0.45 (0.27)	− 1.66	0.110	
6	Peak (standardised by key)	Amount: Lean	Natal family/Bird	0.05 (0.24)	0.21	0.832	84
		Effort: Hard		− 0.55 (0.24)	− 2.32	0.023	
		Sex: M					
7	Peak rate	Amount: Lean	Natal family/Bird	− 0.04 (0.16)	− 0.46	0.646	84
		Effort: Hard		− 0.19 (0.09)	− 2.22	0.035	
		Sex: M		− 0.01 (0.09)	− 0.01	0.927	
		Key: Centre		− 0.19 (0.05)	− 3.64	< 0.001	
		Key: Right		− 0.17 (0.05)	− 3.64	< 0.001

### Effects of developmental experience, individual identity, natal family and sex on timing performance

**Fig. 6:**
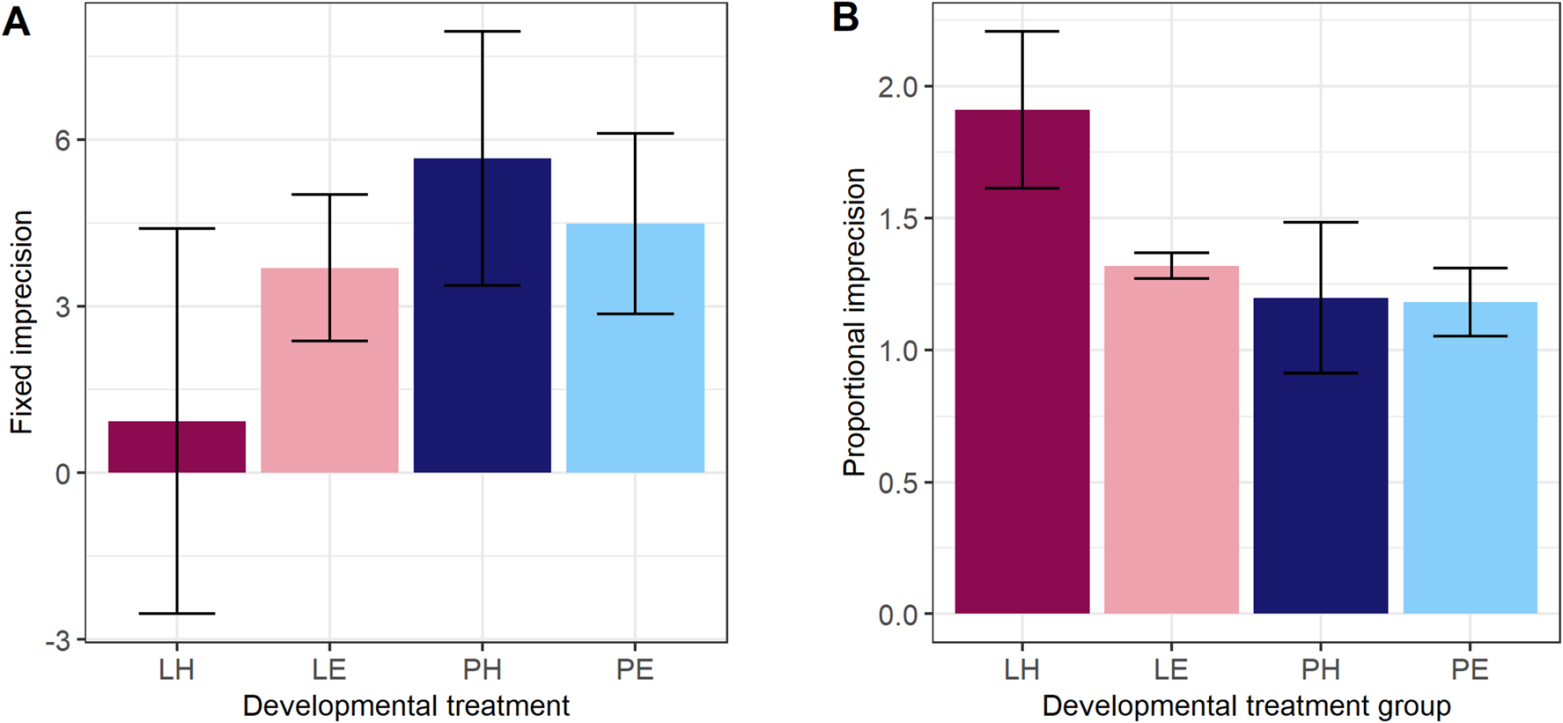
Effects of developmental treatments on timing imprecision. **a** Fixed imprecision. **b** Proportional imprecision. Developmental treatments are Effort: H is Hard, E is Easy; Amount: L is Lean, P is Plenty. Shown are means ± one standard error

We modelled birds’ fixed imprecision and proportional imprecision as outcome variables, with the developmental treatments Amount and Effort, as well as their interaction, as fixed predictors (Table 2 models 3 and 4). Developmental treatments did not significantly predict either fixed imprecision (Fig. 6a) or proportional imprecision (Fig. 6b). Conclusions were unchanged using the truncated version of the fixed imprecision variable instead. However, it is noteworthy that the experimental group that was significantly less impulsive than the others (Lean–Hard) also had the highest average proportional imprecision values (Fig. 6b).

We also carried out exploratory analyses of whether developmental treatments predicted birds’ Peak, Peak Rate or Spread for each key (Table 2 models 5–7). Amount significantly predicted Spread (Table 2, model 5). Across keys, birds raised on a Lean diet had larger Spreads than those raised with Plenty to eat as nestlings (estimated marginal means ± SE: Lean 0.28 ± 0.20, Plenty − 0.27 ± 0.20). Effort significantly predicted Peak across keys (Table 2 model 6). Birds that experienced Hard begging effort as nestlings had earlier Peaks than those that had experienced Easy begging effort (estimated marginal means ± SE: Hard − 0.26 ± 0.15, Easy 0.29 ± 0.16). Effort also significantly predicted Peak Rate (Table 2 model 7). Birds that experienced Easy begging effort as nestlings had a higher Peak Rate than those that had experienced Hard begging effort (estimated marginal means ± SE: Hard 0.51 ± 0.06, Easy 0.70 ± 0.06). There was also a significant effect of key: birds showed higher Peak Rate on the left key than the other two keys (Table 2 model 7; estimated marginal means ± SE: Left key 0.72 ± 0.05, Centre key 0.53 ± 0.05, Right key 0.55 ± 0.05). We found no significant effect of sex on any of the measures of timing performance (Table 2 models 3–7).

There was evidence of individual consistency. When the data from the final 4 days of PROBE trials were split into the penultimate two and last 2 days, individual identity accounted for 60% of the variance in fixed imprecision and 71% in proportional imprecision. In contrast, the variance accounted for by natal family was estimated at zero for both fixed and proportional imprecision.

## Discussion

Our starting point was recent rat findings showing that greater timing imprecision is associated with greater impulsivity (Marshall et al. 2014; McClure et al. 2014; Smith et al. 2015). We first sought, through a numerical model, to establish why this should be the case. Our model highlighted the importance of distinguishing between fixed imprecision (the constant amount of imprecision that applies independently of the delay to be timed) and proportional imprecision (the amount by which imprecision increases as the delay to be timed increases). The model predicted that greater imprecision should increase impulsivity only for fixed imprecision; for proportional imprecision, greater imprecision should in fact reduce impulsivity. We then measured timing imprecision in a cohort of European starlings whose impulsivity had been previously measured. We found, qualitatively supporting the predictions of the model, that greater fixed imprecision tended to associate with greater impulsivity, whilst greater proportional imprecision was associated with less impulsivity.

The reasons that the effects of the two types of imprecision are predicted by the model to be in opposite directions are as follows. Increasing the fixed imprecision means adding a fixed amount of noise to the perception of the duration of both delays. As this is the same amount of extra noise for both, it has a proportionately greater over-valuing effect on the smaller sooner reward, since the discount function is steeper at shorter delays, and hence the impact of any given amount of over- or under-perception is larger. On the other hand, increasing the proportional imprecision means adding an amount of noise to the perception of the delay that is much larger for the longer duration. This larger increment of extra noise overwhelms the fact that the discount function is flatter in the vicinity of the long delay, and hence produces a greater over-valuing effect on the larger later reward relative to the smaller sooner. In short, the marginal impact of increased imprecision on the relative valuation of a smaller sooner and a larger later reward depends entirely on how that imprecision is added: as a fixed constant regardless of delay, or as a scaling factor proportional to the delay.

In our starling study, we separated out, for the first time, the fixed and proportional components of timing imprecision. We were able to do this because the tri-peak temporal reproduction task provides estimates of imprecision for the same individual for three different intervals, allowing us to extract an intercept and a slope for the relationship of imprecision to the fixed interval, and hence separately characterise fixed and proportional imprecision. An important conclusion from our data is that starling timing does not show the scalar variance property. The fact that imprecision increases for long intervals is often taken as an informal test of scalar variance (Lejeune and Wearden 2006; Wearden and Lejeune 2008). Strict scalar variance is a stronger claim though: the ratio of the imprecision to the FI must be constant across FIs, or, equivalently, a regression line of imprecision on FI should pass through the origin. We found this was not so: in our starlings, the ratio of the imprecision to the FI decreased with increasing FI, and the regression line of imprecision on FI had a positive intercept. The conclusions from this starling study about how impulsivity relates to timing imprecision depend very much on which of the two imprecision measures one uses: greater imprecision tends to be associated with greater impulsivity if you use the fixed (intercept, *α*) measure, but it is associated with less impulsivity if you use the proportional (slope, $\beta$) measure. Thus, our starling findings conform to both the assumptions and the predictions of the numerical model.

Are our starling findings consistent with those from the three rat studies (Marshall et al. 2014; McClure et al. 2014; Smith et al. 2015)? They are in the same direction, albeit non-significantly in our case, if we use our fixed timing imprecision measure, but they appear to be in the contrary direction if we use our proportional timing imprecision measure. None of the three previous rat studies separately reports timing imprecision at different durations, nor attempts to distinguish the fixed and proportional components of imprecision. The clear guidance to the field, then, is to consider carefully whether the measure of timing imprecision being used reflects predominantly fixed imprecision, in which case we might predict a positive association with impulsivity; proportional imprecision, in which case we might predict a negative association; or some mixture of the two, in which case predictions are hard to make. We suggest that imprecision measures taken at very short durations are likely to reflect mostly fixed imprecision, whereas those taken at longer durations are likely to reflect mostly proportional imprecision. In our starling data, the imprecision measured by the Spread of the best-fitting polynomial at the longest FI (45 s) was near-perfectly positively correlated with the proportional imprecision; whereas the fixed imprecision was significantly positively correlated with the Spread at 15 s. Thus, whether a single measure of imprecision mainly reflects fixed or proportional imprecision depends how short or long a duration it is measured at. What constitutes a ‘short’ or a ‘long’ duration will depend on the discount rate of the species in question. The study by McClure et al. (2014), for example, measured imprecision at 11 s, which is closer to our medium delay. However, rats have lower discount rates than starlings (Bateson et al. 2015). Thus, our results are consistent with the results of that study, if we assume that the imprecision measure used by McClure et al. in the rats primarily reflected fixed imprecision.

We found that starlings readily learned the distinct FIs associated with each key in the tri-peak procedure task, and were fairly accurate over all in their timing, as indicated by the locations of the peak rates of responding. The point at which the highest rate of responding switched from key to key was very close to the geometric mean of the two FI durations. This matches previous findings with temporal bisection tasks (Platt and Davis 1983). One interpretation of the fact that switching occurs at the geometric rather than arithmetic mean is that birds perform optimal temporal risk assessment (Balci et al. 2009, 2011). That is, they take into account the greater endogenous uncertainty associated with the long delay, and hence switch responding not half way between the two FI durations, but rather at the point where the probability of either FI timing out given their respective uncertainties favours the longer interval (this is at the geometric mean, see Bateson and Kacelnik 1995). There is other, longstanding evidence that starlings take their own timing imprecision into account in making foraging decisions. For example, in deciding when to give up a foraging patch, starlings wait longer after the time at which they expect a food reward for patches where they have experienced longer inter-reward intervals (Brunner et al. 1992).

In addition to testing the predictions of our model concerning the relationship of timing precision to impulsivity, our empirical study provided detailed data on individual variation in timing in the starling. Birds learned the tri-peak procedure readily, and their responses conformed to the expectations of the paradigm. That is, the peck rate on each key on the PROBE trials showed a single peak close to the trained fixed interval, with a rapid rise before and a slightly slower decline after the interval had passed. By separating each individual’s data into two halves (the final 2 days and the 2 days prior to this), we were able to show that individuals were somewhat consistent in both their fixed and proportional imprecision, suggesting that these are stable individual differences. We did not detect any natal-family effects. This result is somewhat surprising given the occurrence of genetic polymorphisms implicated in temporal perception in humans and between-strain differences in rats (Marinho et al. 2018), although we note our relatively small sample of starlings and especially of natal families (eight families were represented). Our birds also all originated from a single population.

We did not find support for our prediction that the same developmental factors that we have shown to affect impulsivity in these birds (namely, Hard begging effort and Lean amount) should also affect timing imprecision. This would follow logically from the claim that a substantial driver of variation in impulsivity is variation in timing imprecision. However, the developmental effects on fixed and proportional imprecision were not significant. Our other exploratory results concerning developmental experience relate to some of our previous findings with this cohort of birds. In the present study, we found that birds which were made to beg harder as nestlings (the Hard treatment) had earlier Peaks, but lower Peak Rates. This is somewhat paradoxical in light of previous findings. Increasing reward value in peak procedure tasks both shifts peak responding earlier, and increases response rates (Galtress and Kirkpatrick 2009). Thus, if the Hard birds valued food rewards more highly, we might expect both earlier Peaks and higher Peak Rates. We have found similarly paradoxical results on these same birds in other behavioural paradigms. The Hard birds better defend their rate of energy intake when foraging is effortful (Neville et al. 2017; Dunn et al. 2018). On the other hand, they also maintain lower body weights, presumably by eating less overall. Here, it seems that their lower Peak Rate, which suggests lower food motivation, patterns with their lower bodyweights; whilst their earlier Peaks pattern with their defence of their rate of energy intake. We also found that birds that experienced restricted early food supply (the Lean treatment) had poorer precision as measured by the Spreads across the individual keys. This would suggest the importance of early nutrition in the development of precise timing.

One limitation of our study was that the choice impulsivity task consisted of one smaller sooner to larger later comparison, whereas previous studies have included multiple smaller sooner delay conditions (Marshall et al. 2014). However, the associations with timing in the study by Marshall et al. (2014) were with preference for smaller sooner averaged across the different smaller sooner delays, Moreover, individuals were highly consistent in their preference across different delays, suggesting that any single delay would be adequate for these purposes. Another limitation of our study is that fixed and proportional imprecision were highly (negatively) collinear. This prevented us entering both terms in the same model to predict impulsivity, which, if possible, would have been preferable as a test of the numerical model. It is possible that this negative dependence is a genuine biological relationship. However, we note that it could be at least partly an artefact of measurement error. With imprecision estimated from just three FIs, there is a substantial amount of measurement error on the slope of the regression of imprecision on FI (i.e. the proportional imprecision). Any measurement error that causes this slope to be measured as steeper than it really is, is also likely to lead to an estimate of the intercept as lower than it really is. Any measurement error that causes the slope to be measured as flatter than it really is will tend to lead to an estimated intercept that is larger than it should be. Thus, the strong negative dependence of fixed and proportional imprecision, which almost guarantees that their respective effects on impulsivity will be in contrary directions (as predicted by the numerical model), could in part be a consequence of coupling due to shared measurement error. Future investigations should measure fixed and proportional imprecision at a greater number of distinct FIs to mitigate this issue.

## Supplementary Information

Below is the link to the electronic supplementary material.

Supplementary file1 (PDF 759 KB)

## References

[CR1] Balci F, Freestone D, Gaillistel D (2009). Risk assessment in man and mouse. Proc Natl Acad Sci U S A.

[CR2] Balci F, Freestone D, Simen P (2011). Optimal temporal risk assessment. Front Integr Neurosci.

[CR3] Bates D, Maechler M, Bolker B, Walker S (2015). Fitting linear mixed-effects models using lme4. J Stat Softw.

[CR4] Bateson M, Brilot BO, Gillespie R (2015). Developmental telomere attrition predicts impulsive decision-making in adult starlings. Proc R Soc B Biol Sci.

[CR5] Bateson M, Kacelnik A (1995). Preferences for fixed and variable food sources: variability in amount and delay. J Exp Anal Behav.

[CR6] Brunner D, Kacelnik A, Gibbon J (1992). Optimal foraging and timing processes in the starling, *Sturnus vulgaris*: effect of inter-capture interval. Anim Behav.

[CR7] Cardinal RN, Aitken MRF (2010). Whisker: a client-server high-performance multimedia research control system. Behav Res Methods.

[CR8] Church RM, Meck WH, Gibbon J (1994). Application of scalar timing theory to individual trials. J Exp Psychol Anim Behav Process.

[CR9] Denny M (2017). The fallacy of the average: on the ubiquity, utility and continuing novelty of Jensen’s inequality. J Exp Biol.

[CR10] Dunn J, Andrews CP, Nettle D, Bateson M (2018). Early-life begging effort reduces adult body mass but strengthens behavioural defence of the rate of energy intake in European. R Soc Open Sci.

[CR11] Dunn J, Andrews C, Nettle D, Bateson M (2019). Developmental history, energetic state and choice impulsivity in European starlings, *Sturnus vulgaris*. Anim Cogn.

[CR12] Evenden JL (1999). Varieties of impulsivity. Psychopharmacology.

[CR13] Feenders G, Bateson M (2013). Hand rearing affects emotional responses but not basic cognitive performance in European starlings. Anim Behav.

[CR14] Galtress T, Kirkpatrick K (2009). Reward value effects on timing in the peak procedure. Learn Motiv.

[CR15] Gibbon J (1977). Scalar expectancy theory and Weber’s law in animal timing. Psychol Rev.

[CR16] Gibbon J (1991). Origins of scalar timing. Learn Motiv.

[CR17] Hinton S, Meck W (1997). How time flies: functional and neural mechanisms of interval timing. Adv Psychol.

[CR18] Jensen J (1906). Sur les fonctions convexes et les inégalités entre les valeurs moyennes. Acta Math.

[CR19] Lejeune H, Wearden JH (2006). Scalar properties in animal timing: conformity and violations. Q J Exp Psychol.

[CR20] Marinho V, Oliveira T, Rocha K (2018). The dopaminergic system dynamic in the time perception: a review of the evidence. Int J Neurosci.

[CR21] Marshall AT, Smith AP, Kirkpatrick K (2014). Mechanisms of impulsive choice: I. Individual differences in interval timing and reward processing. J Exp Anal Behav.

[CR22] Matell MS, Bateson M, Meck WH (2006). Single-trials analyses demonstrate that increases in clock speed contribute to the methamphetamine-induced horizontal shifts in peak-interval timing functions. Psychopharmacology.

[CR23] Mazur JE (2004). Risky choice: selecting between certain and uncertain outcomes. Behav Anal Today.

[CR24] Mazur JE, Biondi DR (2009). Delay-amount tradeoffs in choices by pigeons and rats: hyperbolic versus exponential discounting. J Exp Anal Behav.

[CR25] McClure J, Podos J, Richardson HN (2014). Isolating the delay component of impulsive choice in adolescent rats. Front Integr Neurosci.

[CR26] Moeller FG, Barratt ES, Dougherty DM (2001). Psychiatric aspects of impulsivity. Am J Psychiatry.

[CR27] Nettle D, Andrews CP, Reichert S et al (2017) Early-life adversity accelerates biological ageing: experimental evidence from the European starling. Sci Rep 40794 10.1038/srep40794 PMC5240102 28094324

[CR28] Neville V, Andrews C, Nettle D, Bateson M (2017). Dissociating the effects of alternative early-life feeding schedules on the development of adult depression-like phenotypes. Sci Rep.

[CR29] Patros CHG, Alderson RM, Kasper LJ (2016). Choice-impulsivity in children and adolescents with attention-deficit/hyperactivity disorder (ADHD): a meta-analytic review. Clin Psychol Rev.

[CR30] Paule MG, Meck WH, Millan DEMC (1999). The use of timing behaviors in animals and humans to detect drug and/or toxicant effects. Neurotoxicol Teratol.

[CR31] Platt JR, Davis ER (1983). Bisection of temporal intervals by pigeons. J Exp Psychol Anim Behav Process.

[CR32] R Core Development Team (2018) R: a Language and Environment for Statistical Computing

[CR37] R Development Core Team (2011) R: A Language and Environment for Statistical Computing

[CR33] Smith AP, Marshall AT, Kirkpatrick K (2015). Mechanisms of impulsive choice: II. Time-based interventions to improve self-control. Behav Process.

[CR34] Verdejo-García A, Lawrence AJ, Clark L (2008). Impulsivity as a vulnerability marker for substance-use disorders: review of findings from high-risk research, problem gamblers and genetic association studies. Neurosci Biobehav Rev.

[CR35] Wearden JH, Lejeune H (2008). Scalar properties in human timing: conformity and violations. Q J Exp Psychol.

[CR36] Wittmann M, Paulus MP (2008). Decision making, impulsivity and time perception. Trends Cogn Sci.

