# Supplementary material for: Time perception and patience: individual differences in interval timing precision predict choice impulsivity in European starlings, Sturnus vulgaris
Source: Anim Cogn. 2021 Jan 12;24(4):731–45. doi: 10.1007/s10071-020-01456-2 (PMC8238733; doi:10.1007/s10071-020-01456-2)
Supplement: Supplementary file 1 — Supplementary file1 (PDF 759 KB) [file 10071_2020_1456_MOESM1_ESM.pdf]

Supplementary Information

Figure S1. Violation of scalar variance property.

Data show the ratio of the Spread to the fixed interval duration for each bird at each fixed interval. Under the scalar variance property, this ratio should be the same for all three fixed intervals.

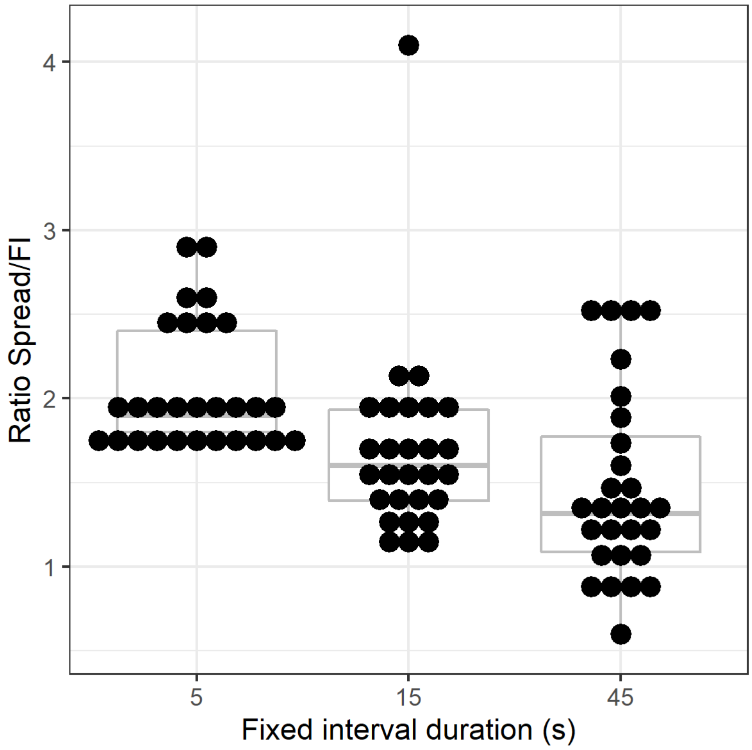

Figure S2. Interval timing performance of individual starlings

Data shown for each bird are mean points for PROBE trials during the final 4 days of the tri-peak procedure task, with the fitted 10th order polynomial (solid line). Dashed reference lines show the trained FI durations (SHORT, 5s in red; MEDIUM, 15 s in green; LONG, 45 s in blue). Pecks to each of the keys are coloured red for lefthand key (trained on the 5 s FI), green for centre key (15 s FI) and blue for righthand key (45 s FI). Individual starling identity is denoted by a 4 letter code above each panel A to BB.

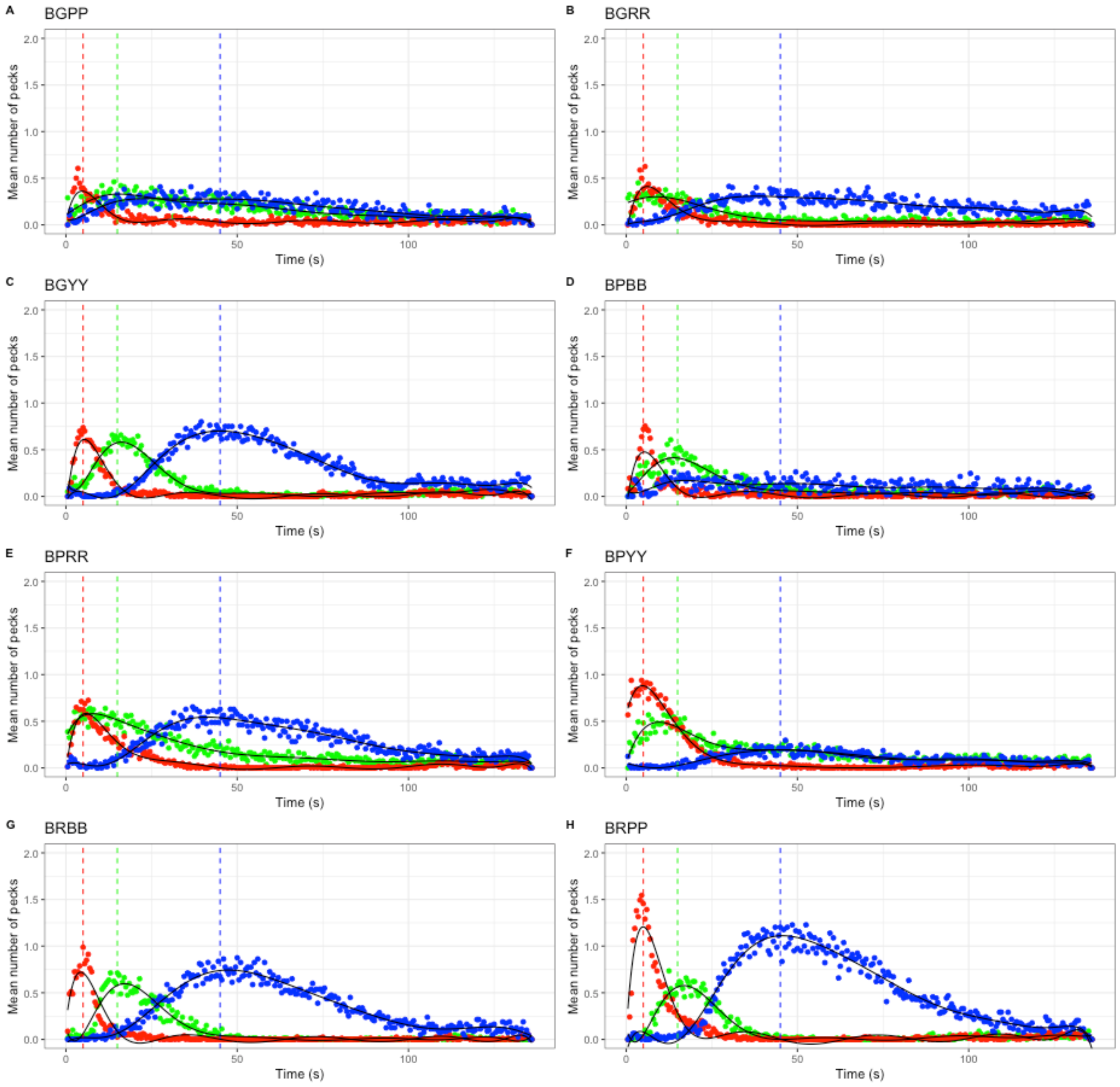

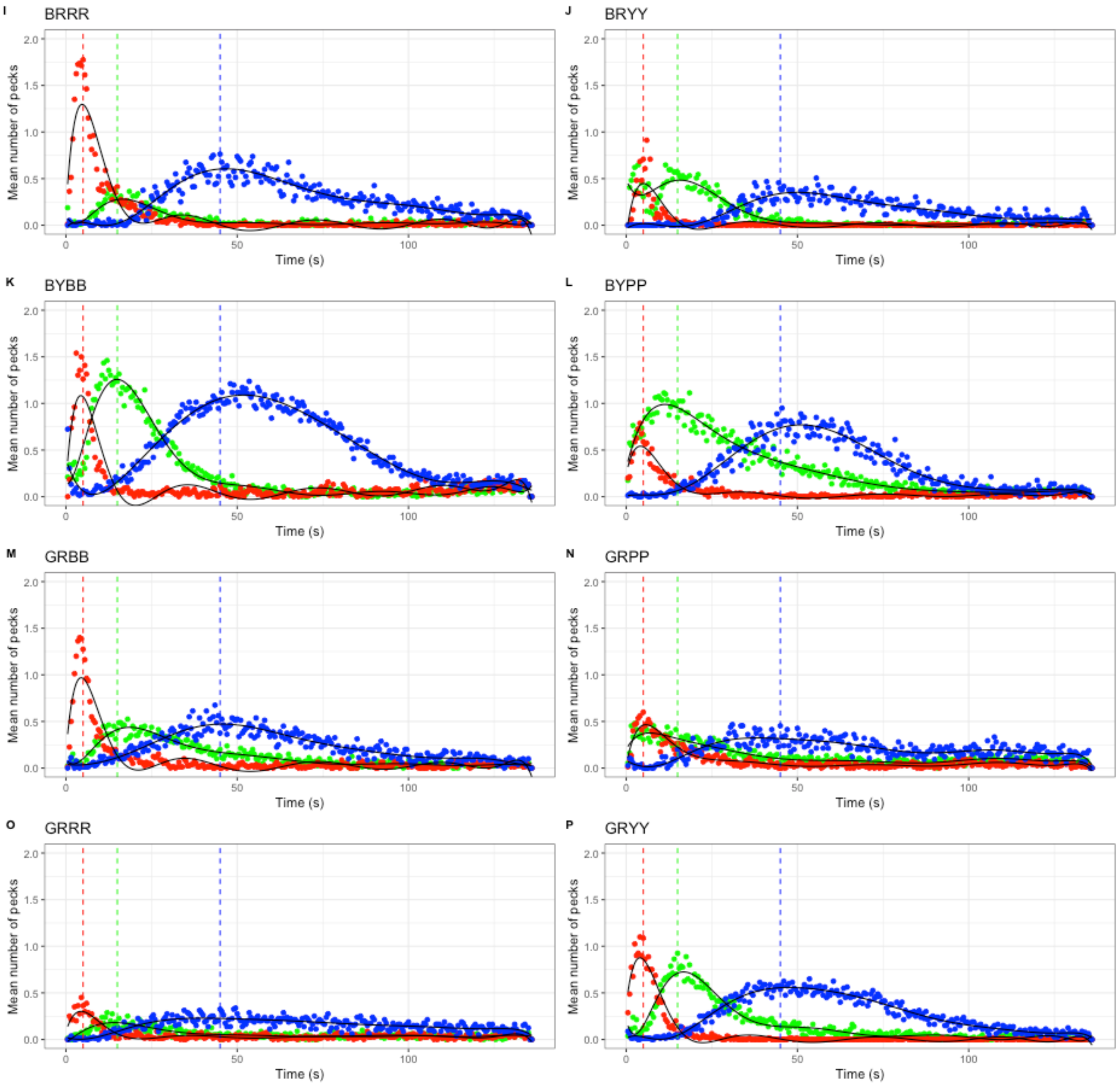
I
BRRR
J
BRYY
K
BYBB
L
BYPP
M
GRBB
N
GRPP
O
GRRR
P
GRYY
Mean number of pecks
Time (s)
0.0
0.5
1.0
1.5
2.0
0
50
100

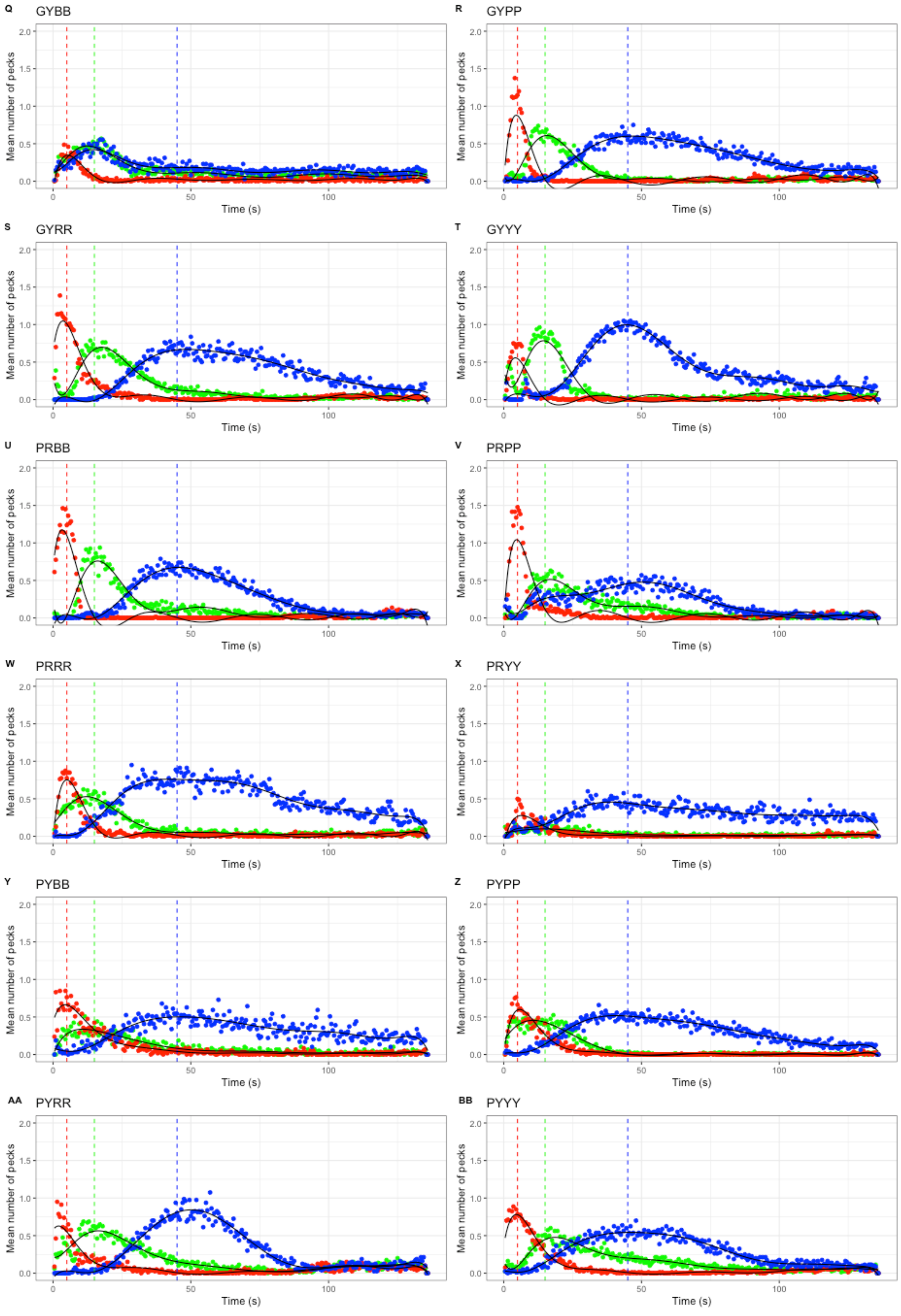

Q GYBB
R GYPP
S GYRR
T GYYY
U PRBB
V PRPP
W PRRR
X PRYY
Y PYBB
Z PYPP
AA PYRR
BB PYYY
Mean number of pecks
0.0
0.5
1.0
1.5
2.0
Time (s)
0
50
100
